# Structural Insights Into How Proteoglycans Determine Chemokine-CXCR1/CXCR2 Interactions: Progress and Challenges

**DOI:** 10.3389/fimmu.2020.00660

**Published:** 2020-04-24

**Authors:** Krishna Rajarathnam, Umesh R. Desai

**Affiliations:** ^1^Department of Biochemistry and Molecular Biology, The University of Texas Medical Branch at Galveston, Galveston, TX, United States; ^2^Sealy Center for Structural Biology and Molecular Biophysics, The University of Texas Medical Branch at Galveston, Galveston, TX, United States; ^3^Department of Microbiology and Immunology, The University of Texas Medical Branch at Galveston, Galveston, TX, United States; ^4^Department of Medicinal Chemistry, Institute for Structural Biology, Drug Discovery and Development, Virginia Commonwealth University, Richmond, VA, United States

**Keywords:** glycosaminoglycan, proteoglycan, chemokine, nuclear magnetic resonance, structure, heparan sulfate, chondroitin sulfate, heparin

## Abstract

Proteoglycans (PGs), present in diverse environments, such as the cell membrane surface, extracellular milieu, and intracellular granules, are fundamental to life. Sulfated glycosaminoglycans (GAGs) are covalently attached to the core protein of proteoglycans. PGs are complex structures, and are diverse in terms of amino acid sequence, size, shape, and in the nature and number of attached GAG chains, and this diversity is further compounded by the phenomenal diversity in GAG structures. Chemokines play vital roles in human pathophysiology, from combating infection and cancer to leukocyte trafficking, immune surveillance, and neurobiology. Chemokines mediate their function by activating receptors that belong to the GPCR class, and receptor interactions are regulated by how, when, and where chemokines bind GAGs. GAGs fine-tune chemokine function by regulating monomer/dimer levels and chemotactic/haptotactic gradients, which are also coupled to how they are presented to their receptors. Despite their small size and similar structures, chemokines show a range of GAG-binding geometries, affinities, and specificities, indicating that chemokines have evolved to exploit the repertoire of chemical and structural features of GAGs. In this review, we summarize the current status of research on how GAG interactions regulate ELR-chemokine activation of CXCR1 and CXCR2 receptors, and discuss knowledge gaps that must be overcome to establish causal relationships governing the impact of GAG interactions on chemokine function in human health and disease.

## Introduction

Proteoglycans (PGs), a diverse class of forty three members, are expressed by most cell types and tissues and play vital roles in human physiology and disease (1–5). PGs are classified into four classes: cell-surface, pericellular, extracellular, and intracellular (1). Sulfated glycosaminoglycans (GAGs) are a family of linear polysaccharides covalently attached to core proteins, and are the glycan part of PGs (6). Mammals express five sulfated GAGs that differ in their backbone structure and sulfation pattern: heparin, HS, CS, DS, and KS. Cell surface PGs consist of two classes – transmembrane (TM) and glycosylphosphatidylinositol (GPI) PGs, and both carry HS and/or CS chains (7, 8). Pericellular and extracellular PGs are secreted and exist as macromolecular complexes with other PGs and proteins (1, 9, 10). Pericellular PGs predominantly carry HS, and extracellular PGs predominantly carry CS (1). Intracellular PG consists of only one member, serglycin, which serves as a storage for granule proteins. Whereas serglycin in mast cells carries predominantly heparin, serglycin in platelets carries CS and DS (1, 11, 12).

A PG can have one single GAG chain or hundreds of GAG chains, and one or two types of GAGs; additionally, the prevalence of the individual GAGs can vary, with HS and CS being the most preferred. GAGs are intrinsically heterogeneous due to differential sulfation and epimerization. Therefore, the complexity of PG structures arise not only from their amino acid sequence, size, shape, and the type and number of attached GAG chains, but also from the diversity in GAG structures. PG physiology is equally diverse and includes both structural and functional roles that are highly context-dependent. Whereas their structural role as a component of the ECM and PCM in providing cellular and tissue integrity and stability is relatively well understood, their functional roles – as a platform for binding hundreds of proteins of various classes, from chemokines and growth factors to proteases – are less well understood.

Humans express around 47 chemokines that share the following fundamental properties. They are around 70 to 100 amino acids in length, have the same tertiary structure consisting of three β-strands and an α-helix stabilized by disulfide bonds, exist reversibly as monomers and dimers and occasionally as higher order oligomers, bind GAGs, activate receptors of the GPCR (G protein-coupled receptor) class, and mediate trafficking of various immune and non-immune cells to distal and remote locations (13–15). Fundamental to any given chemokine or cell type, chemokine function must be highly regulated so that the right number of cells reach their target site at the right time to elicit the right response. For instance, seven human chemokines – CXCL1, CXCL2, CXCL3, CXCL5, CXCL6, CXCL7, and CXCL8, all characterized by the conserved N-terminal “ELR” motif – are agonists for CXCR1 and CXCR2 receptors. Whereas all ELR-chemokine monomers are potent CXCR2 agonists, ELR-chemokine dimers are differentially active for CXCR2, and CXCL8 monomer alone functions as a potent CXCR1 agonist. CXCR1 and CXCR2 are expressed in diverse cell types, including neutrophils that combat infection, oligodendrocytes in spinal cord patterning, and neuronal cells that regulate pain, in addition to regulating trafficking and proliferation of cancer cells (16–19). In this review, we focus on the structural basis and molecular mechanisms by which GAGs regulate ELR-chemokine activation of CXCR1 and CXCR2 receptors that are expressed on neutrophils and other cell types. Insights from this subset of chemokines and cell type reflect the current status and challenges for the GAG interactions of the entire chemokine family at large.

To appreciate the diversity in PG structures, we discuss syndecans as an example. Syndecans are cell surface PGs, and vertebrates express four syndecans: syndecan-1, -2, -3, and -4 (3, 20, 21). Whereas syndecan-4 (Sdc-4) is widely expressed in most tissues, Sdc-1 is expressed on epithelial and endothelial cells, and Sdc-2 and Sdc-3 are expressed selectively in cells of mesenchymal and neuronal origin, respectively. Studies using *Sdc-1* and *Sdc-4* KO mice and cell-based assays show that Sdc-1 and Sdc-4 regulate chemokine-mediated neutrophil recruitment (22–29). Chemokine binding to syndecans has been implicated in the activation of signaling pathways, changes in actin cytoskeleton, downregulation of gap junction proteins, and an increase in permeability (30–32). Reorganization of endothelial PGs also facilitates neutrophil adhesion, an early critical step that precedes crossing the endothelium (33). However, Sdc-1 and Sdc-4 are of varying molecular weights, show low amino acid sequence homology in their ectodomains, and whereas Sdc-1 carries 3 HS and 2 CS chains, Sdc-4 carries only 3 HS chains. HS chains are located close to the N-terminus at the distal end of the ectodomains; therefore, HS-bound GAGs are more accessible for interacting with infiltrating leukocytes. CS chains are closer to the membrane surface, and thus CS-bound chemokine could play a prominent role in mediating signaling events in the endothelium. From biophysical studies that have shown chemokine binding can induce crosslinking and clustering of GAGs, it has been proposed chemokine binding to GAGs can result in clustering and reorganization of the glycocalyx triggering endothelial signaling (34).

The basic building block of HS consists of repeating disaccharide units of D-glucuronic acid (GlcA) and N-acetyl-D-glucosamine (GlcNAc). HS has a modular structure, with sulfated sequences separated by non-sulfated regions, and is also more diverse due to differential N-sulfation and O-sulfation. In addition to N-sulfation, glucosamine can have 6-O sulfation, and GlcA can have 2-O sulfation and epimerize at C5 to L-iduronic acid (IdoA). The basic building block of CS consists of D-GlcA and N-acetyl-D-galactosamine (GalNAc), and on average, the disaccharide unit has one sulfate with O-sulfation either at C4 or C6 of GalNAc. Knowledge of the manner in which differences in CS and HS structures and fine structure of a given GAG impact chemokine binding and function is necessary.

In response to infection or injury, ELR-chemokines released by resident cells at the site of insult navigate across the epithelium, ECM, and endothelium to the vasculature, and provide directional cues by establishing haptotactic and chemotactic gradients that direct neutrophils to the insult site (35–39). GAG interactions impact most aspects of chemokine function, including chemokine blood levels, lifetime by preventing proteolysis and being washed away with blood flow, makeup of chemokine gradients, and chemokine presentation to the receptor on neutrophils (37–45). During an inflammatory response, neutrophils are observed in as early as an hour and can last over a day or more; chemokine levels during this period can vary by orders of magnitude as a function of time and space (46–48). The chemokine concentration is higher close to the membrane surface due to GAG interactions, and fall steeply toward the center of the vessel; it is also higher at sites where neutrophils extravasate into the tissue due to small dimensions of the capillaries and high numbers of endothelial venules.

**FIGURE 1:**
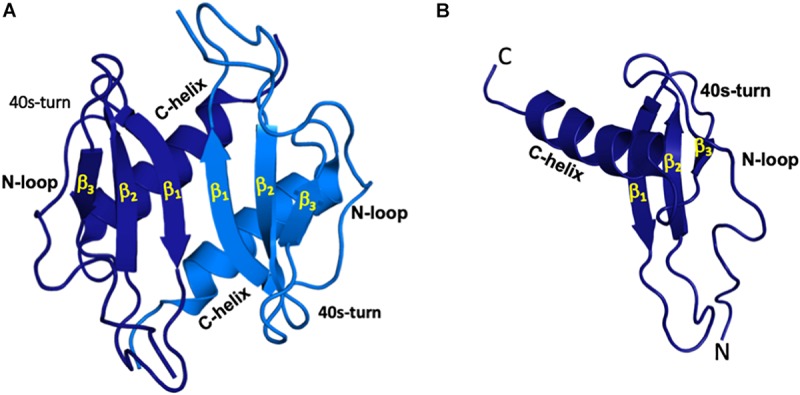
All ELR-chemokines share the same structural fold. Structures of CXCL5 dimer **(A)** and monomer **(B)** as a representative of ELR chemokines are shown. The individual monomers in the dimer are shown in dark and light blue for clarity and different GAG-binding regions (N-terminal loop, 40s turn, and C-terminal α-helix) are labeled.

ELR-chemokines reversibly exist as monomers and dimers (Figure 1), and it is well established that the dimeric form binds GAG with higher affinity (49–55). At any given time and location, all four forms – monomer and dimer in solution and in GAG-bound forms – must exist in dynamic equilibrium. However, their relative amounts will depend on chemokine and GAG concentrations and four equilibrium constants – between monomer and dimer, monomer and GAG, dimer and GAG, and monomer-bound GAG and dimer-bound GAG. Engineered trapped monomers and dimers in animal models show distinctly different neutrophil recruitment profiles, indicating that the monomer-dimer equilibrium regulates recruitment (56–59). These observations indicate that chemokine dimerization and GAG interactions control chemotactic and haptotactic gradients that are coupled to the flux, duration, and kinetics of circulating neutrophil egress to the tissue. In disease situations, dysregulation in chemokine expression and/or disruption in monomer/dimer ratio could lead to either low or uncontrolled neutrophil trafficking resulting in unresolved inflammation and significant collateral tissue damage and disease.

**FIGURE 2:**
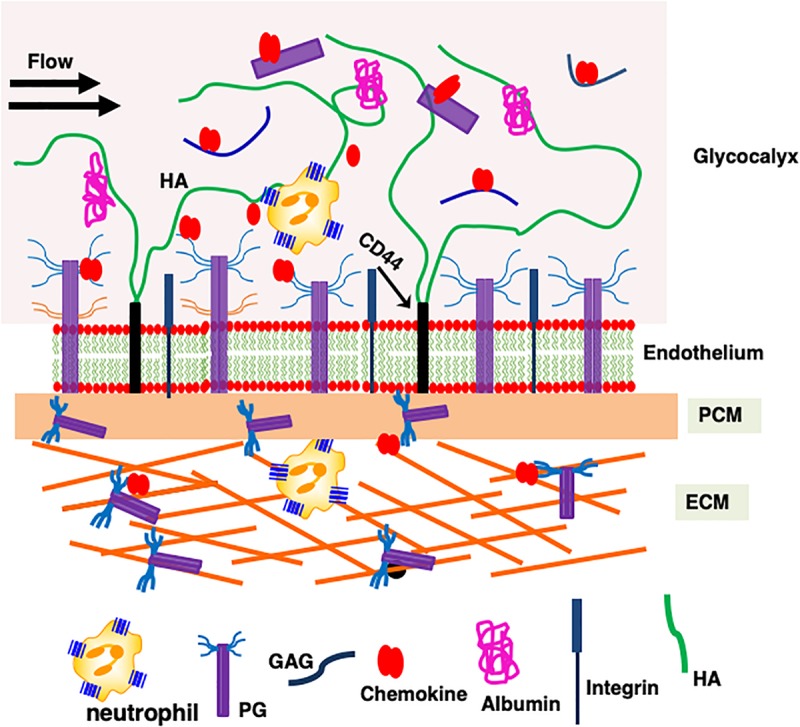
A schematic showing how proteoglycan interactions regulate chemokine-mediated neutrophil trafficking. Endothelial glycocalyx consists of a number of secreted PGs and various proteins such as albumin that functions as a barrier between blood flow and the endothelium. Critical components of the endothelium and glycocalyx are labeled. This figure is not drawn to scale, and its purpose is to illustrate different anatomical regions that are involved in chemokine-mediated neutrophil recruitment to the insult site.

Neutrophil trafficking is orchestrated by the interaction of ELR-chemokines with the endothelial, ECM, PCM, and glycocalyx PGs (Figure 2). Chemokines released at the site of insult first encounter ECM PGs, then cross the rather thin PCM located on the abluminal side of the endothelium, and then the endothelium before interacting with the endothelial cell surface PGs (60). Chemokines then enter the glycocalyx, which is a specific form of PCM as it functions as an interface between the endothelial cell surface and the blood flow (61). The dimensions of the glycocalyx are much larger (∼1000 nm) in comparison with the dimensions of a chemokine (∼3 nm), syndecan ectodomain (<100 nm), or a typical GAG (∼80 nm) (62). The glycocalyx, besides several PGs and proteins, also contains the non-sulfated GAG HA, and syndecan ectodomain and free HS generated upon cleavage by bacterial and endogenous proteases during an inflammatory response (4, 63–66). HA, which can be a few microns long, plays an important role in keeping the components of the glycocalyx together. From the perspective of chemokine interactions, endothelial PGs and the glycocalyx must be considered as a continuum rather than as two distinct compartments, and GAG interactions at this location are crucial as they set the stage for the subsequent steps of neutrophil trafficking to the target site. Chemokines then finally diffuse into the vasculature, where they encounter neutrophils before being washed away with the blood flow.

**FIGURE 3:**
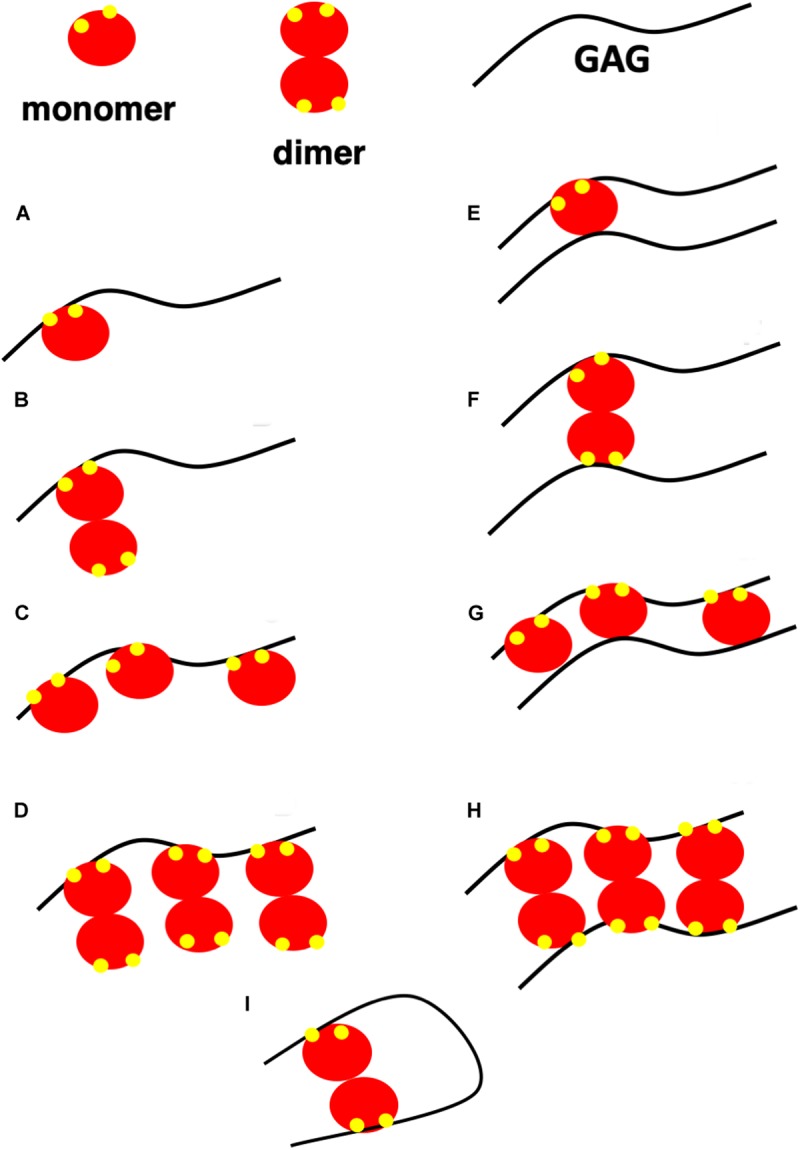
A schematic showing possible GAG-bound ELR chemokine structures. In this schematic, a GAG corresponds to heparin or HS, monomer and dimer corresponds to CXCL5 monomer and dimer, and GAG-binding residues are shown in yellow. **(A,B)** A single chemokine monomer or dimer binding a single GAG occurs at low chemokine concentrations. With increasing concentration, dimer-bound GAG is favored due to higher binding affinity. **(C,D)** Chemokines bind GAGs like beads on a string at high chemokine concentrations. Of the two, model **(D)** is favored due to higher binding affinity of the dimer. **(E–H)** Chemokines bind two GAGs within a PG or between PGs. Of the different models, models E and G are unlikely as a monomer has only one GAG binding site. **(I)** Horseshoe model of HS binding to a chemokine dimer. HS structure consists of sulfated regions (NS) interspersed with non-sulfated regions (NA). HS is of the form NS-NA-NS in the horseshoe model.

Understanding how PG interactions impact chemokine function requires knowledge of the molecular mechanisms and the structural basis, such as binding-interface residues, geometry, affinity, and stoichiometry of both monomer and dimer binding to different GAG types, as well as how PG structures and distribution of GAGs in a given PG impact chemokine binding interactions. There is no evidence that chemokines bind to the protein component of the PGs, and so we confine our discussion to binding to GAGs. A schematic of different GAG-chemokine complexes that could exist during neutrophil recruitment at different locations is shown in Figure 3. At low concentrations, essentially a single chemokine monomer or dimer will bind each GAG chain (models A and B). GAGs are long linear polysaccharides; therefore, when chemokines are in excess, multiple chemokines – either as monomers or dimers – can bind a single GAG, like beads on a string (models C and D). Model C will be sparsely populated as chemokines form dimers at higher concentrations, and dimers bind GAG with higher affinity. Because most proteoglycans carry two or more GAG chains, binding could also occur due to proximity effects, as shown in models E to H. HS has also been proposed to adopt horseshoe geometry (model I) due to its modular structure (67).

## Molecular Basis of Heparin Interactions

The molecular basis of the binding of human CXCL1, CXCL5, CXCL7, and CXCL8 and of mouse KC/mCXCL1 and MIP2/mCXCL2 to heparin oligosaccharides has been characterized using solution NMR spectroscopy (53–55, 68–72). GAGs are acidic, and ELR-chemokines are basic, indicating that electrostatic and H-bonding interactions play a major role in driving the binding process. NMR chemical shifts of the backbone amides are sensitive to their environment; therefore binding-induced chemical shift changes were used to identify the GAG-binding interface residues, which were then used in generating models of the chemokine-GAG complex using HADDOCK docking software. These studies used oligosaccharides from a disaccharide to a 26 mer, and the results indicated that an octasaccharide or longer is necessary for capturing the binding interface. Shorter oligosaccharides were ineffective in identifying all of the binding residues due to weaker binding, because binding affinity decreases with decreasing size. These studies also indicated that (i) dimerization and GAG binding are coupled and that the dimer binds GAG with higher affinity; (ii) the binding-interface is extensive and plastic; (iii) electrostatic and H-bonding interactions mediate affinity and specificity; (iv) lysines (Lys) dominate over arginines (Arg) at the binding interface; and (v) GAG-binding geometries differ among chemokines.

## Gag-Binding Signature and Binding Interactions

**FIGURE 4:**
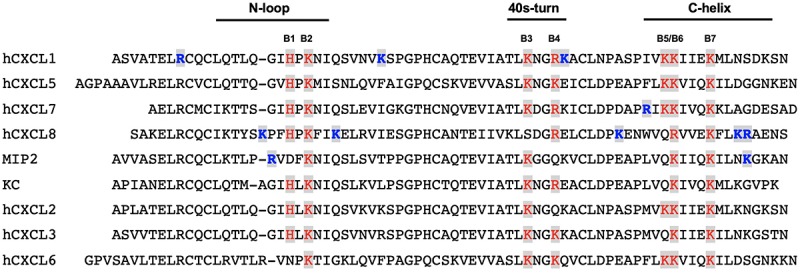
GAG-binding residues in human and mouse ELR-chemokines. A GAG-binding residue is labeled as conserved if present in five or more of the seven human sequences. Conserved GAG-binding residues are labeled B1 to B7 and are in red. Binding residues unique to a given chemokine are in blue.

As ELR chemokines share a similar structural fold, any differences in GAG interactions must be due to differences in the amino acid sequence. NMR, mutagenesis, and *in vivo* neutrophil trafficking studies have shown that GAG binding is driven by conserved and chemokine-specific lysine, arginine, and histidine residues located in the N-loop, 40s turn, and C-terminal helix (Figure 4). A GAG-binding residue is labeled as conserved if present in five or more of the seven human sequences. Conserved GAG-binding residues, labeled B1 to B7, are in red. Binding residues that are not conserved and unique to a given chemokine are in blue. Whereas CXCL1, CXCL7, and CXCL8 have unique GAG-binding residues, CXCL5 has none. The large numbers of unique GAG-binding residues in CXCL1 and CXCL8 are noteworthy. In CXCL1 alone, an N-terminal arginine is involved in binding, and this arginine is absolutely conserved and is critical for receptor activation in all ELR-chemokines (66). Therefore this residue, from a GAG-binding perspective, is labeled as a specific and not a conserved residue. GAG interactions for CXCL2, CXCL3, and CXCL6 are not known. Sequences of CXCL2 and CXCL3 are highly similar to CXCL1 but are missing a basic residue at positions B4 and B5, respectively, and CXCL6 is also distinct as it is missing B3. Residues B2, B3, B6, and B7 are involved in binding in both mouse chemokines (KC/mCXCL1 and MIP2/mCXCL2). However, MIP2 is missing B1 and B4, but has two unique GAG-binding residues whereas KC has none. These observations collectively suggest that each member of ELR-chemokines has a unique GAG-binding signature by differential combination of conserved and chemokine-specific residues.

## Geometry of Binding

**FIGURE 5:**
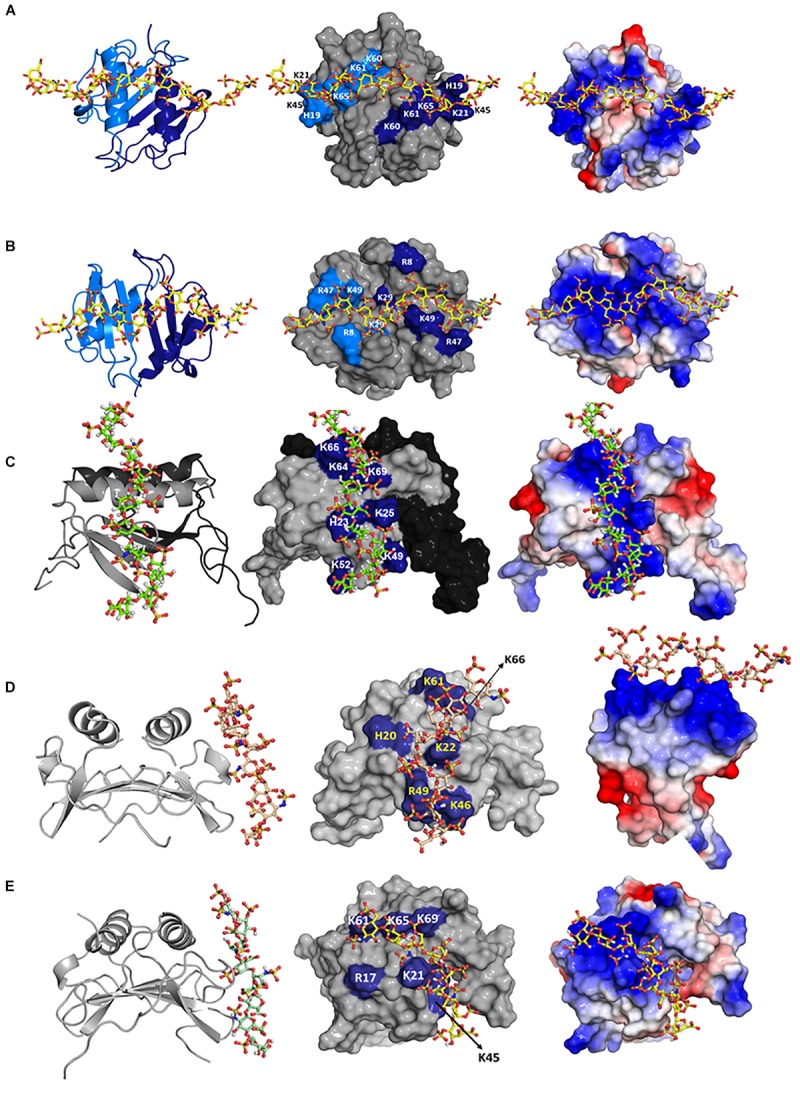
Structural models of heparin bound to **(A)** CXCL1 α-domain, **(B)** CXCL1 β-domain, **(C)** CXCL5, **(D)** KC/mCXCL1, and **(E)** MIP2/mCXCL2. Left column: chemokine dimer structures are shown in ribbon presentation and heparin as sticks. Middle column: Heparin binding residues are highlighted on a space-filling model. Right column: Heparin binding residues are shown as the electrostatic surface. In panels **(A,B)**, two monomers of the dimer are shown in light and dark blue (left column) and heparin-binding residues from both monomers are highlighted in light and dark blue (middle column). In panel **(C)**, two monomers of the dimer are shown in gray and black (left and middle columns), and GAG-binding residues are labeled only in the gray monomer. In panels **(D,E)**, both monomers are shown in gray, and only one monomer of the dimer is labeled.

Mapping heparin-binding residues on chemokine structures suggests multiple binding geometries. Therefore, to gain more definitive insights into the binding geometry, we generated structures of heparin-bound chemokine complexes using HADDOCK modeling software, which uses residues implicated in binding from NMR chemical shift changes as ambiguous restraints, shape complementarity, and energetics to drive the docking process (73). Three different HADDOCK runs were performed to ensure that the input constraints did not bias specific structural models, and that all possible binding geometries within a monomer and across the dimer interface were considered – binding of one heparin with constraints given to both monomers of the dimer, binding of two heparins with constraints given to both monomers of the dimer, and binding of one heparin with constraints given to only one monomer of the dimer. Models generated from these studies for CXCL1, CXCL5, KC, and MIP2 are shown in Figure 5. For CXCL1, modeling indicates two heparin chains span the dimer interface that are located on opposite faces of the protein (defined as the α-domain and β-domain). For CXCL5, modeling suggests two models, of which only one model (model-I; in which CXCL5 binds within the monomer) is consistent with all of the NMR data. In the second model, CXCL5 binds across the dimer interface and does not involve 40s turn residues (model-II). Isothermal titration calorimetry studies indicate two heparins bind per CXCL5 dimer that are consistent only with Model-I (71). Modeling studies for KC/mCXCL1 and MIP2/mCXCL2 also suggest two binding geometries, one within the monomer and one across the dimer interface. NMR data are consistent only with model-I, and ITC data also indicate a stoichiometry of two heparins per dimer (54). Modeling studies for CXCL8 suggest three binding geometries within a monomer and one across the dimer interface (74). Additional studies are required to determine whether binding occurs across the dimer or within the monomer. Modeling studies for CXCL7 also suggest two binding geometries, one within the monomer and one across the dimer interface (53). Additional studies are required to unambiguously define whether binding occurs via one or both interactions.

## Stoichiometry and Thermodynamics

Knowledge of the stoichiometry is essential not only to validate GAG-binding geometry but also to understand whether a GAG-bound chemokine can bind its receptor. Unlike protein-protein and protein-DNA complexes, defining the stoichiometry for GAG-chemokine complexes is not straightforward. The stoichiometry can vary because GAGs are linear polysaccharides consisting of repeating disaccharide units resulting in multiple binding sites (Figure 2). Further, the stoichiometry and the number of species will vary with GAG size, chemokine:GAG ratio, and experimental conditions such as pH and ionic strength. Commonly used techniques such as SPR and fluorescence spectroscopy report a binding constant and cannot provide insights into the stoichiometry. ITC, in addition to providing a binding constant and thermodynamic signatures (enthalpy and entropy), also provides stoichiometry (75). ITC has additional advantages: it is quite sensitive, does not require modification of the protein or GAG, and can measure binding constants from the micromolar (μM) to nanomolar (nM) range. Interestingly, despite similar binding affinities, thermodynamic signatures for KC and MIP2 are quite different. Molecular dynamic (MD) simulations also indicate striking differences in binding interactions at a residue-specific level, and suggest that binding interactions are not additive but coupled, and that the binding of any given residue is governed by the binding interactions of all residues.

## Molecular Basis of Hs Interactions

**FIGURE 6:**
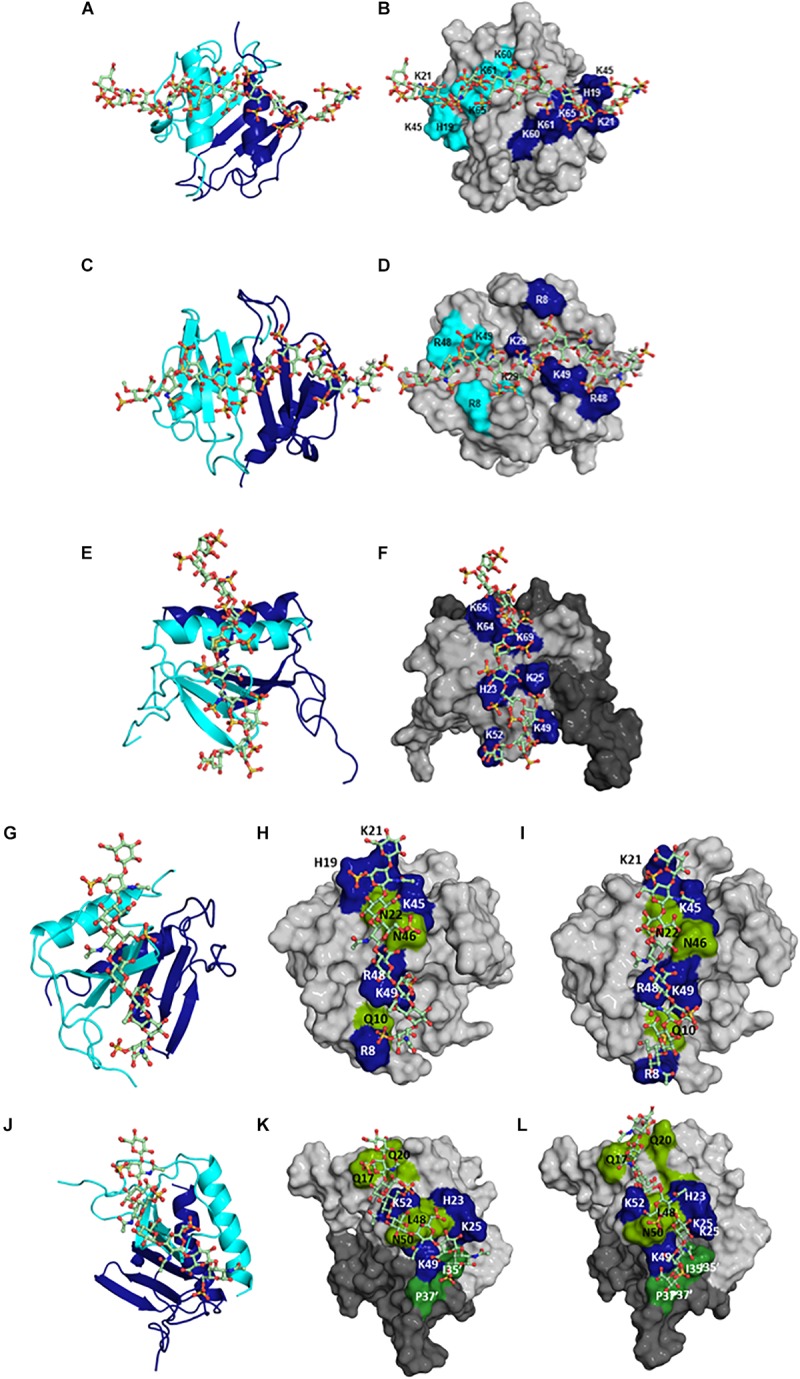
Structural models of HS bound to **(A,B)** CXCL1 α-domain, **(C,D)** CXCL1 β-domain, and **(E,F)** to CXCL5; of CS bound to **(G,H)** CXCL1 γ-domain and to **(J,K)** CXCL5; of DS bound to **(I)** CXCL1 γ-domain and to **(L)** CXCL5. In panels **(A,C,E,G,J)**, CXCL1 and CXCL5 dimer structures are shown in ribbon presentation and GAG as sticks. Two monomers of the dimer are shown in blue and cyan. In panels **(B,D,F,H,I,K,L)**, GAG binding residues are highlighted on a space-filling model. In panels **(F,K,L)**, two monomers of the dimer are colored in light and dark gray. In panels **(H–K)**, polar residues are in green. In panels **(K,L)**, residues from the second monomer are distinguished by the ′ symbol. HS binds across the dimer interface in CXCL1 and within the monomer in CXCL5; CS and DS bind within a monomer in CXCL1 and bind both monomers of the dimer in CXCL5.

In the context of *in vivo* function, chemokines interact with HS and not heparin. Both HS and heparin share a repeating disaccharide unit composed of glucosamine and hexuronic acid. Heparin is preferred for structural and biophysical studies as it is more uniformly sulfated and because of the availability of size-defined oligosaccharides. Heparin is assumed to be a good surrogate for describing HS interactions; this could be an oversimplification considering that their solution structures are different, and the fine structure of HS due to differential sulfation cannot be captured by heparin (76–78). We recently characterized the binding of heparin and HS polymers to CXCL1 and CXCL5 using NMR spectroscopy (79). Binding-induced chemical shift changes for HS were similar to heparin, indicating that the same basic residues mediate binding both GAGs and that their binding geometry is the same (Figures 5, 6). Because HS fine structure arises due to differential N- and O-sulfation, HS variants that are either missing N-sulfate, 2-O sulfate, or 6-O sulfate were modeled for binding both chemokines. The binding geometries of all variants were similar to that of heparin, suggesting that the binding interface is plastic, and that differences in sulfation do not impact the binding geometry (79). In principle, a single HS polymer could bind both binding sites as a horseshoe (Figure 3), but NMR studies cannot distinguish between two HS chains binding two binding sites or a single HS binding both sites. This is the first NMR study for any protein that has characterized residue-specific binding interactions to heparin and HS polymers, and it validates the premise that heparin is a good surrogate at least for describing structural features such as the binding interface. Whether this is universally applicable to all HS binding proteins remains to be determined.

## Molecular Basis of Cs and Ds Interactions

Very little is known regarding the structural basis of the binding interactions of CS, DS, or KS, or how the distribution of basic residues in a given protein mediate binding different GAGs. To address this knowledge gap, we characterized the binding of CS and DS oligosaccharides to CXCL1 and CXCL5 using NMR spectroscopy (79). DS can be considered a variant of CS, with the difference arising from C5 epimerization of GlcA to IdoA. For both chemokines, the dimer compared to the monomer bound CS and DS with higher affinity similar to what was observed for HS, the binding affinities were lower than for HS, and binding interactions were quite different from HS. For both chemokines, chemical shift changes were observed for N-loop and 40s turn basic residues, but not for the helical residues, which is quite striking, as helical residues in ELR-chemokines have long been implicated as important in GAG binding. For CXCL1, CS and DS bind a set of basic resides that lie within the monomer (defined as the γ-domain) in contrast to two HS chains spanning the dimer interface that are located on opposite faces of the protein (Figure 6). For CXCL5, in addition to perturbation of the N-loop and 40s turn basic residues, several N-loop and 30s loop non-basic residues were also perturbed, and modeling studies indicated a binding geometry across the dimer interface (Figure 6). CS exists in two forms with a sulfate at either 4-O or 6-O position, and modeling studies indicate that their binding interactions are similar (79). In contrast to CXCL1 and CXCL5, an NMR study has reported that the binding interactions of CS, DS, and heparin to CXCL8 are similar (80). Collectively, these data demonstrate how differences in the participation of a few basic residues and GAG backbone structure and sulfation pattern can result in diverse binding interactions.

## Molecular Basis of Proteoglycan Gag Interactions

**FIGURE 7:**
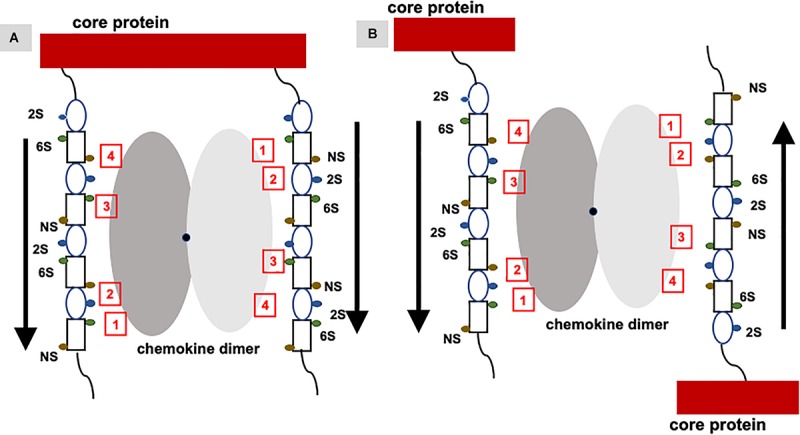
Binding of a chemokine dimer to PG GAGs. Monomers of the chemokine dimer are shown in different shades of gray. For illustrative purposes, GAG-binding residues in each monomer of the chemokine dimer are labeled 1 to 4, which run in opposite directions because of two-fold symmetry about the dimer interface (shown by a black dot). Schematic of an heparin octasaccharide with iduronate and glucosamine shown as an oval and rectangle, and N-sulfate (NS), 2-sulfate (2S), and 6-sulfate (6S) shown as spheres in different colors. Helical structure of heparin clusters the 3 sulfate groups on opposite faces of the helical axis (44). **(A)** Binding of a chemokine dimer to two GAG chains within a PG. Direction of the GAG orientation are shown by arrows. The schematic shows GAG interactions of the two monomers cannot be the same, and that binding to both GAG chains is only possible if the binding interactions of the two monomers are different. **(B)** Binding of a chemokine to two GAG chains from two PGs. Direction of the GAG orientation are shown by arrows. In this case, two monomers of the dimer can encounter GAG chains running in opposite directions and so similar interactions to both monomers are possible. Such binding could occur in PCM and ECM due to some degree of motional freedom of the PGs.

Studies described so far have characterized chemokine binding to free GAGs; the question therefore arises to what extent these studies capture binding to *in vivo* PG GAGs. PG GAG chains are assembled on serine residues in core proteins by a series of glycosyltransferases and modification enzymes in the Golgi; therefore all GAG chains have the same orientation within a PG, and further, their mobility is restricted compared to free GAGs due to its covalent linkage to the core protein. Biophysical, cellular, and *ex vivo* studies have shown that a single GAG can bind multiple chemokines, binding promotes chemokine accumulation and GAG crosslinking, and that *in vivo* binding occurs at distinct anatomical sites (34, 41, 81–83). A chemokine dimer can bind a single GAG chain, bind two GAG chains within a PG, and/or two GAG chains from different PGs (Figure 7). For a chemokine dimer to bind two GAG chains within a PG, the dimensions of the chemokine dimer must be compatible to the distance between the GAGs. However, GAG binding sites in the chemokine dimer are antiparallel due to two-fold symmetry, and so GAG binding at the second site will not occur unless GAG interactions are non-specific. Molecular modeling studies indicate that GAGs show a preferred directionality (polarity). Binding to two GAGs within a PG can occur if the binding surface in two monomers of the dimer are different and are therefore not restricted by symmetry considerations. A chemokine dimer can bind two GAGs from two different PGs as it is not restricted by symmetry considerations. Further, structures of the GAG chains in the same PG are not necessarily equivalent or have to be perfectly aligned as GAGs are dynamic and flexible. Fluorescence recovery after photobleaching (FRAP) experiments have shown CXCL12 dimer binding can induce crosslinking of HS chain (84). In this setup, HS chains are immobilized and so they have the same orientation. NMR studies show heparin binds across the CXCL12 dimer interface suggesting a stoichiometry of one GAG per dimer (85). Therefore, how CXCL12 is able to crosslink HS chains is not clear, suggesting the structural basis of binding cell surface GAGs could be more complex. Experiments specifically designed to address chemokine binding to PG GAGs both in the context of cell surface and ECM environments are essential to address this critical missing knowledge.

## Can a Gag-Bound Chemokine Form a Ternary Complex With Its Receptor?

**FIGURE 8:**
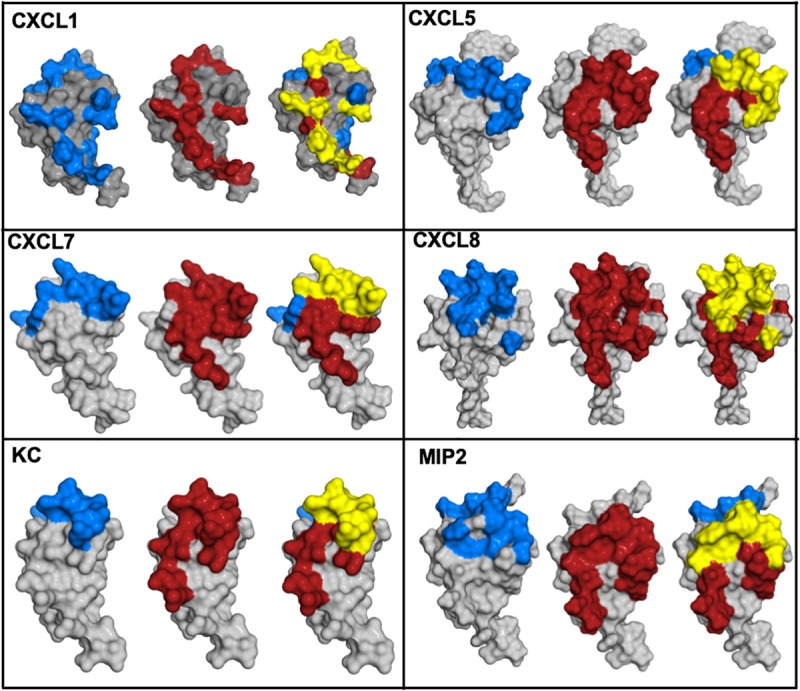
Chemokine structures showing GAG and receptor binding regions. Receptor-binding domains are in red, GAG-binding domains are in blue, and residues that are common to both are in yellow.

**FIGURE 9:**
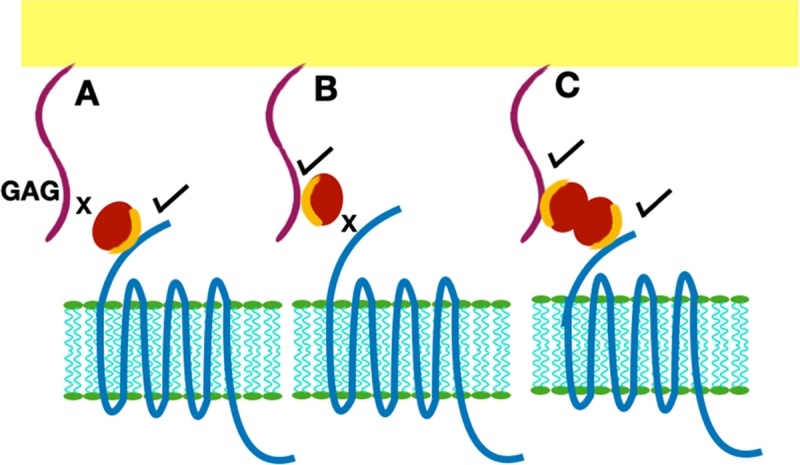
Presentation of GAG-bound chemokine for receptor interactions. Chemokine is shown as a red sphere, and shared residues for GAG and receptor binding are shown in orange. **(A)** Chemokine monomer bound to the receptor cannot bind the GAG. **(B)** Chemokine monomer bound to a GAG chain cannot bind the receptor. **(C)** A chemokine dimer bound to a single GAG chain. In this case, one monomer can bind the GAG and the second monomer of the dimer is available for receptor interactions.

**FIGURE 10:**
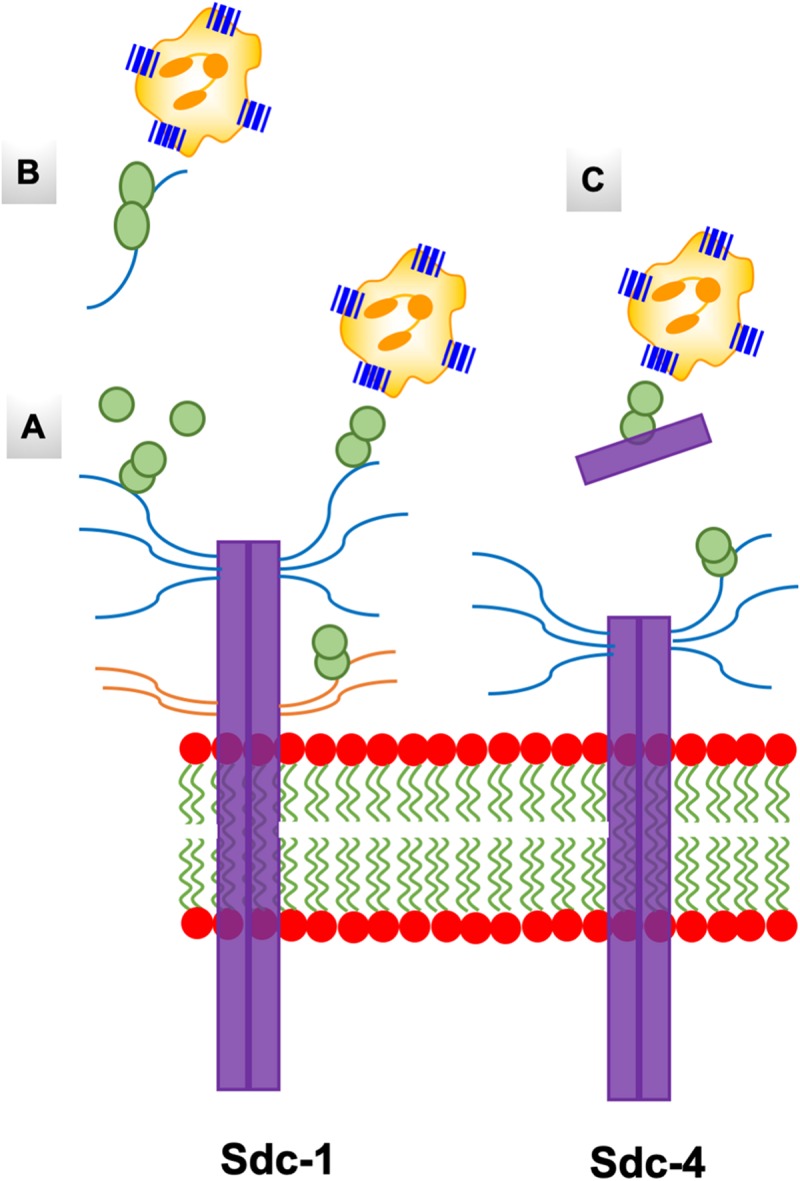
A schematic of the endothelial syndecans and glycocalyx. Syndecans span the membrane and exist as dimers. Sdc-1 is larger (33 kDa) compared to Sdc-4 (22 kDa), and the extracellular domains carry HS and CS chains that are shown in blue and orange, respectively. Chemokine dimer bound to HS chain on syndecans **(A)**, and to free HS and syndecan ectodomain in the glycocalyx **(B,C)** are accessible to the CXCR2 receptor on neutrophils.

Functional studies show receptor activation involves interactions between the chemokine N-loop/β_*3*_-strand and receptor N-domain residues (defined as Site-I) and between the ligand N-terminal and receptor extracellular/transmembrane residues (Site-II) (16, 86, 87). For all ELR-chemokines, some of the N-loop residues involved in GAG binding are also involved in receptor binding (49, 68–72, 74, 79, Figure 8), indicating that GAG interactions disrupt receptor binding. NMR experiments have shown that a GAG-bound monomer cannot form a ternary complex due to occlusion of receptor-binding residues, indicating only the free monomer can activate the receptor (74, 88). Similarly, residues that mediate receptor interactions are occluded in a chemokine dimer sandwiched between GAGs, and a ternary complex cannot be formed. For a chemokine dimer bound to a single GAG, the second receptor binding site is available for receptor interactions, which is the case when the chemokine dimer is bound at the edge of HS chains in syndecan (Figures 9, 10). This model will only apply if GAG binding occurs within a monomer; therefore, HS-bound CXCL1, or a chemokine in which HS binds both monomers of the dimer like a horseshoe cannot bind the receptor. Both Sdc-1 and Sdc-4 carry HS chains, and Sdc-1 also carries CS chains that are located close to the membrane; therefore, CS-bound chemokines are likely to be less important for CXCR2 activation. ECM and PCM PGs and free HS and cleaved PG ectodomains in the glycocalyx are not restricted by constraints of the membrane, and a chemokine dimer bound to a single GAG chain can therefore bind the receptor (Figure 10). However, direct experimental evidence in support of GAG-bound dimer binding the receptor is lacking. CC chemokines, in addition to dimers, also form oligomers and polymers. However, their dimer interactions involve N-loop residues, and so dimers and oligomers cannot activate the receptor. CC oligomers also bind GAG with higher affinity, and so it is very unlikely that receptor binding residues are accessible in the GAG-bound form. Recently, it has been proposed that GAG-bound chemokine regulates a “cloud” of solution phase chemokines within the glycocalyx (labeled as “chemokine cloud” model), and that it is this soluble form for any chemokine that interacts with leukocyte-bound receptors (89). Our model is in agreement with the “chemokine cloud” model for the ELR chemokine monomer but not necessarily for the dimer. Structural features of ELR chemokine dimer allows GAG-bound dimer to interact with the receptor (Figures 9, 10). The availability of well-characterized trapped dimeric and monomeric chemokines, such as those used in the NMR studies, would be most useful in designing experiments that address how chemokine dimers bind PG GAGs and whether PG GAG-bound chemokine can access the receptor (53, 54, 74).

## Specificity of Gag-Binding Residues

A characteristic feature of ELR-chemokines is the preference of GAG-binding residues for Lys over Arg (Figure 4). In general, arginines are enriched relative to lysines in protein-protein and protein-DNA Interfaces (90, 91). The pKa of the surface Lys amino and Arg guanidinium groups are >10, indicating that they are always protonated and charged at physiological pH. Whereas the Lys NH${}_{3}^{+}$ group is symmetric and has a simple structure, the guanidinium group of Arg is planar and asymmetric with a more complex structure. The guanidinium group, compared to the NH${}_{3}^{+}$ group, can form stronger electrostatic interactions with the sulfate anion (92). To understand the preference for lysines, three of the lysines in CXCL8 known to be involved in GAG interactions were mutated to arginine. The Lys to Arg mutants bound heparin with higher affinity as expected, but interestingly, exhibited highly impaired neutrophil recruitment activity in a mouse model (93). Mutating these lysines to alanine results in reduced GAG affinity and also reduced neutrophil recruitment (59). These data make a compelling case that the specificity imparted by a lysine cannot be replaced by an arginine, and that enhanced GAG binding as a result of arginine substitution is actually detrimental, and most likely impacts one or more GAG-related functions, which in turn impairs neutrophil recruitment function.

$${\text{LysNH -C}}_{\text{α}}{\text{H -C}}_{\text{β}}{\text{H}}_{\text{2}}{\text{ -CγH}}_{\text{2}}{\text{ -C}}_{\text{δ}}{\text{H}}_{\text{2}}{\text{ -C}}_{\text{ε}}{\text{H}}_{\text{2}}{\text{-N}}_{\text{ζ}}{\text{H}}_{\text{3}}^{\text{+}}$$

$${\text{ArgNH -C}}_{\text{α}}{\text{H -C}}_{\text{β}}{\text{H}}_{\text{2}}{\text{ -C}}_{\text{γ}}{\text{H}}_{\text{2}}{\text{ -C}}_{\text{δ}}{\text{H}}_{\text{2}}{\text{ -N}}_{\text{ε}}{\text{H-C}}_{\text{ζ}}{\text{= (N}}_{\text{η}}{\text{H}}_{\text{2}}{\text{)}}_{\text{2}}^{\text{+}}$$

## Future Directions and Challenges

We contend that the current knowledge on GAG interactions is just the “tip of the iceberg,” and there is still much to be learnt if we were to compare to what is known for protein-protein and protein-DNA interactions. In particular, knowledge of how individual residues and their crosstalk with other binding residues mediate GAG interactions, the role of dynamics, and the relationship between structure, dynamics, thermodynamics, and function is lacking. NMR, ITC, and MD studies of KC and MIP2 binding to heparin highlight the importance of such knowledge for deeper and more quantitative insights (54). GAGs and Lys and Arg side chains are conformationally dynamic (94–96), and very little is known regarding how dynamic characteristics of the protein and GAG drive binding interactions. NMR studies tailored to probe lysine NH${}_{3}^{+}$, arginine guanidinium, and histidine imidazole groups can provide crucial insights that are not accessible based on backbone chemical shifts (69, 93, 97). Chemical shifts of the lysine NH${}_{3}^{+}$ group are not observed in the free protein due to rapid exchange with the solvent, but are observed in the bound form due to its interaction with GAG acidic groups; these shifts are more sensitive than backbone chemical shifts in defining whether a particular lysine is involved in binding or not (69). Side chain chemical shifts are essential for characterizing side chain dynamics, and such studies have proven to be quite useful for describing the properties of the individual lysines in protein-DNA complexes (96). The histidine imidazole side chain can exist in three forms due to two titratable nitrogens. NMR chemical shifts of the conserved histidine (B2) in CXCL1 and CXCL8 indicate that the structural state and their GAG interactions are different (97), emphasizing differences in chemokine-specific and residue-specific interactions that could not have been inferred from any other experiment. There is evidence that GAG binding is also driven by H-bonding interactions mediated by polar residues such as asparagine (Asn) and glutamine (Gln) (98, 99). NMR and MD studies of several ELR chemokines suggest Asn and Gln are involved in GAG interactions (54, 100). Considering these residues form weak H-bonds compared to lysines and arginines, the impact of mutating these residues on affinity, geometry, and neutrophil trafficking should give definitive insights into whether they play a role in defining specificity or affinity or both.

Much less is known regarding how GAG backbone structure and distribution of sulfates and carboxylates dictate binding. NMR-based structural models provide such knowledge but need to be independently validated. Whereas measuring binding-induced chemical shifts of protein GAG-binding residues is straightforward, the reciprocal experiment where binding-induced changes in GAG chemical shifts or assigning it to a specific sulfate and carboxylate is not trivial. Knowledge of the structures of GAG-bound chemokine complexes allows describing which sulfate and carboxylate interacts with which protein residue. However, structure determination of GAG-protein complexes, either by crystallography or NMR spectroscopy, faces many challenges, including lack of crystals suitable for crystallography and isotopically labeled homogeneous GAGs for NMR methods. This is evident if we consider that of the more than 100,000 structures deposited in the protein data bank (PDB), only around 100 correspond to those of protein-GAG complexes. The majority of these structures correspond to select proteins such as proteases and growth factors bound to small heparin oligosaccharides. As far as chemokines go, a crystal structure of chemokine CCL2 bound to a heparin octasaccharide, and an NMR structure of CCL5 bound to CS have been reported (101, 102). Homogeneous heparin and HS oligosaccharides have been produced by chemoenzymatic methods by a few select labs (103–105), but are not routinely available for researchers. Glycan Therapeutics (106), founded by academic researchers with the aid of a STTR grant from the NIH, recently announced it will make homogeneous GAGs including ^13^C-labeled oligosaccharides commercially available. These will be a major boon not only for structural studies using NMR and X-ray methods but also for studies focused on understanding how GAG structural features impact binding interactions and function.

Considering that there are tens of thousands of possible structures of, say, an HS octasaccharide, synthesis of all HS variants or their characterization is not realistic. However, in principle, their interactions can be characterized *in silico*. In recent years, much effort has gone into this arena, including developing energy functions, MD and docking programs, and user-friendly software that can be accessed by the GAG community at large (107–112). Promising variants from this exercise could then be tested by experimental methods. Advances in structural and computational methods will also have a major impact for GAG-based therapeutics. Heparin is used extensively as an anticoagulant, and several heparin-based drugs have shown protection in human diseases (113–115). Heparin (unfractionated, low molecular weight and other derivatives) has been investigated in clinical trials for various forms of inflammation (116–119), although it is not clear which sequences, if any, would offer the best response. A recent discovery that a HS hexasaccharide selectively targets cancer stem cells is exciting (120). Though HS is known to engage diverse mitogenic factors, this observation suggests that one or more HS oligosaccharides could display selectivity for a specific chemokine and thereby exhibit a distinct anti-inflammatory phenotype. However, heparin-based drugs have their own limitations, including intrinsic heterogeneity, potential CS contamination that could be detrimental, and an unknown mode of action *in vivo*, as it binds a number of proteins (121). Hydrogels based on heparin and heparin derivatives were also shown to outperform the standard-of-care product Promogran, by their ability to scavenge chemokines, and effectively reducing neutrophil activity and alleviating disease symptoms in humans (122). Therefore, advances in understanding GAG interactions will also be useful for designing GAG-based drugs that could have high clinical relevance for treating a number of human pathologies.

## Conclusion

There is now compelling evidence that GAG interactions and binding geometries can be quite diverse for closely related chemokines, indicating that GAG interactions are highly specific and that this information is coded in the chemokine sequence. Chemokines were first reported around 30 years ago, and the fact that they are ligands for GPCRs and bind GAGs was reported a few years later. Studies characterizing chemokines and/or related to chemokines continue to be an area of active research, which is evident if we consider a PubMed search for the word “chemokine” results in >100,000 hits, with >5000 hits for 2019 alone. Though all ELR-chemokines activate CXCR2, animal models and clinical data provide convincing evidence that they are not redundant and are selectively and differentially expressed in different tissues for eliciting diverse physiological roles. We propose that differences in GAG interactions play important roles in defining the unique *in vivo* phenotype of each chemokine. Continued advances in structural, biophysical and computational methods for characterizing protein-GAG and protein-PG complexes, and the availability of homogeneous GAGs should lead to significant expansion of the knowledge base on the many ways through which these fascinating biopolymers orchestrate function in human pathophysiology.

## Author Contributions

KR conceived and wrote the manuscript with input and suggestions from UD. Both authors approved the final version of the manuscript.

## Conflict of Interest

The authors declare that the research was conducted in the absence of any commercial or financial relationships that could be construed as a potential conflict of interest.

## Abbreviations

CS: chondroitin sulfate
CXCL: CXC ligand
CXCR2: CXC chemokine receptor 2
DS: dermatan sulfate
ECM: extracellular matrix
GAG: glycosaminoglycan
HA: hyaluronic acid
HS: heparan sulfate
ITC: isothermal titration calorimetry
KS: keratan sulfate
MD: molecular dynamics
NMR: nuclear magnetic resonance
PCM: pericellular matrix
PG: proteoglycan
Sdn: syndecan
SPR: surface plasmon resonance.
